# Investigation of Physiological Responses of Different Soybean Cultivars Under Drought Stress

**DOI:** 10.3390/plants15050714

**Published:** 2026-02-27

**Authors:** Yegyeong Bae, Amit Ghimire, Minju Lee, Minsu Jeong, Minju Kwon, Yoonha Kim

**Affiliations:** 1 Department of Applied Biosciences, Kyungpook National University, Daegu 41566, Republic of Korea; 2 Department of Integrative Biology, Kyungpook National University, Daegu 41566, Republic of Korea; 3 Upland Field Machinery Research Center, Kyungpook National University, Daegu 41566, Republic of Korea; 4 Institute of Basic Science Convergence, Kyungpook National University, Daegu 41566, Republic of Korea

**Keywords:** drought, fatty acid, roots, soybean, photosynthetic traits, water use efficiency

## Abstract

Soybeans with modified fatty acid compositions are widely used to improve oil quality and develop functional food products; however, physiological responses to drought stress during early growth stages remain insufficiently understood. This study compared shoot and root responses of three soybean cultivars with contrasting fatty acid profiles: Osoy (elevated linolenic acid; OS), PE529 (elevated oleic acid; PE), and Pungsannamul (PS) under drought stress conditions. Drought significantly reduced plant height, shoot biomass, and leaf area in all cultivars, although the magnitude of reduction differed among genotypes. PS exhibited the greatest decreases in plant height (39%), shoot dry weight (47%), and leaf area (78%) compared with well-watered conditions. In addition, PS showed relatively higher net carbon assimilation rate and stomatal conductance during the early phase of drought, but significantly lower values than OS and PE after 7–8 days of treatment, indicating a relatively higher sensitivity to drought stress. Root trait responses varied among cultivars. Total root length was largely maintained under drought conditions; however, all cultivars exhibited increased root distribution in deeper soil layers. Notably, PE showed a relatively higher proportion of roots at 40 cm depth. Whole-plant water use efficiency (wWUE) did not differ among cultivars under drought conditions; however, Pearson correlation analysis revealed strong associations between wWUE and root traits in PE, including total root length (*r* = 0.72), average root diameter (*r* = −0.77), and root volume (*r* = 0.65). Overall, PE exhibited relatively stable morphological and physiological responses under drought stress, suggesting a comparatively higher adaptive potential during early growth stages.

## 1. Introduction

Soybean (*Glycine max* L.) is a globally important oilseed crop widely utilized for diverse industrial and nutritional purposes. Soybean oil is composed of saturated and unsaturated fatty acids, among which oleic acid (OA), linoleic acid, and α-linolenic acid (ALA) are key determinants of oil quality, oxidative stability, and human health benefits [1]. Because the composition of these unsaturated fatty acids strongly influences the functional and nutritional value of soybean oil, extensive breeding efforts have focused on developing functional cultivars such as elevated-OA and elevated-ALA lines [2,3,4,5]. However, despite the rapid progress in improving these functional traits, foundational studies examining how such cultivars perform physiologically and agronomically under real-world growing conditions remain limited.

With the increasing frequency and intensity of drought events under climate change, maintaining stable soybean productivity has become a major challenge worldwide [6]. Drought stress commonly reduces root biomass, thereby limiting water uptake, and suppresses shoot expansion, leaf area development, and biomass accumulation. Physiologically, drought reduces stomatal conductance, photosynthesis, and transpiration, while altering water-use efficiency [7,8,9]. Beyond these growth and physiological responses, drought has also been reported to influence key seed quality traits, including oil concentration and fatty acid composition [5,10]. Water deficit can change the fatty-acid composition of soybean seeds, but the specific pattern of these changes differs depending on the cultivar and the severity of water stress [11,12]. Understanding how the fatty acid composition of functional soybean cultivars responds or adapts to stress environments is therefore gaining importance from both agronomic and breeding perspectives.

The germination and early seedling stages, in particular, represent a critical window that determines subsequent drought responsiveness and physiological stability [13]. Lipids stored in soybean seeds are predominantly in the form of triacylglycerols (TAG), which serve as the primary energy source during germination and early seedling growth [14]. Upon germination, TAGs are hydrolyzed by lipases into fatty acids, which are subsequently converted to sugars through β-oxidation and the glyoxylate cycle, supporting cellular expansion and division [14,15]. In TAGs containing linoleic acid, a specific linoleate-13-lipoxygenase is capable of oxygenating esterified linoleate without prior lipolysis, and oxygenated fatty acids are preferentially released and oxidized. Thus, genotypes with higher linoleic acid concentration may possess an advantage during germination through this preferential lipid breakdown pathway [16,17]. Indeed, several studies have reported associations between seed fatty acid composition and germination or early seedling performance in various crops. Higher proportions of polyunsaturated fatty acids are often linked to faster germination or improved emergence under low-temperature conditions, suggesting that fatty acid composition can influence early-stage stress sensitivity [15,18,19]. However, most existing studies have focused on cold, osmotic, or heat stress, and the effects of differences in fatty acid composition on early seedling responses under water-limited conditions remain largely unexplored. In other words, experimental evidence on how functional soybean cultivars with contrasting fatty acid profiles respond to drought during early growth is still very limited. This highlights the need to evaluate how differences in fatty acid composition interact with drought stress to shape early morphological and physiological responses.

To reliably compare drought responses among cultivars, experimental conditions must allow strict environmental control. In field environments, maintaining uniform drought intensity is inherently challenging due to variable rainfall [20]. Considering the limitations, the present study employed a controlled-environment pot experiment conducted in a confined growth volume rather than under field conditions.

A commercial horticultural substrate was used as the growth medium because it is free from most pathogens, pests, and weed seeds, and offers homogeneous physical properties. Unlike mineral soils, soilless substrates contain high organic matter and distinct pore structures, resulting in water retention characteristics and matric potential dynamics that differ substantially from soil [21]. In mineral soils, available water is commonly defined as the water held between field capacity (approximately −10 kPa to −33 kPa) and the permanent wilting point (−1500 kPa). In contrast, soilless substrates are described using a different concept for water availability, as proposed by de Boodt and Verdonck [22], reflecting their distinct physical and hydraulic properties. According to this concept, easily available water is defined as the water held between −1 and −5 kPa, while the water buffering capacity corresponds to the volumetric proportion of water released between −5 and −10 kPa, enabling physiological adjustment of plants to declining water potential. In peat-based substrates, the range from −1 to −10 kPa is therefore generally considered plant-available water [22,23], with approximately −10 kPa often used as a theoretical wilting threshold for soilless-grown crops [24]. Because substrate matric potential changes rapidly during drying, continuous monitoring is required to maintain a consistent drought level. However, direct measurement of matric potential is often constrained by the slow hydraulic equilibration between soilless media and sensors, making it difficult to capture rapid moisture changes typical of container-grown systems. To address these limitations, volumetric water content (VWC) was monitored using a frequency-domain reflectometry (FDR) sensor, which measures water content based on dielectric properties and allows rapid detection of changes in substrate moisture [25]. This approach enabled consistent maintenance of target drought levels and reliable comparison of drought responses among functional soybean cultivars.

Given the rising demand for functional soybean cultivars and the projected increase in drought frequency under future climates, understanding how cultivars with contrasting fatty acid compositions respond to water-limited conditions has become increasingly important. Therefore, the present study investigated differences in early shoot and root growth and photosynthetic responses among functional soybean cultivars with distinct fatty acid profiles under drought stress. The findings aim to improve understanding of drought adaptation mechanisms in functional soybeans and provide fundamental insights for future breeding strategies that consider both oil quality improvement and abiotic stress tolerance.

## 2. Results

### 2.1. Volumetric Water Content by Soil Depth

**Figure 1:**
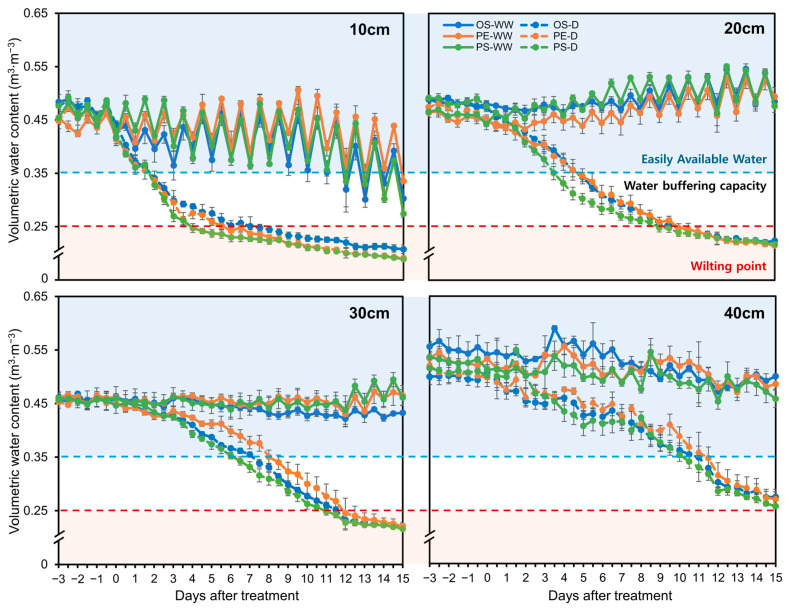
Volumetric water content measured at four soil depths (10, 20, 30, and 40 cm) in OS, PE, and PS cultivars under well-watered (WW) and drought (D) conditions. Measurements were taken from 3 days before treatment initiation (annotated by −3, −2, −1; corresponding to 3, 2, and 1 days prior to day 0) through 15 days after treatment. The light blue region represents the easily available water range, and the light orange region indicates the wilting point. The data were collected in three replicates and were presented as the average ± standard error.

Under well-watered (WW) conditions, soil water content at all depths except 10 cm remained above the WW threshold of 0.35 m^3^∙m^−3^ throughout the experiment. In contrast, under drought conditions, the uppermost layer (10 cm) was the first to drop below 0.25 m^3^∙m^−3^, the threshold for the wilting point, followed sequentially by the 20 cm, 30 cm, and 40 cm layers (Figure 1). Notably, the 40 cm depth consistently maintained higher water content compared to the upper soil layers during the entire experimental period.

To determine whether soil moisture status differed among cultivars at the time of harvest (15 DAT), VWC at each soil depth under drought treatment was statistically analyzed (Supplementary Figure S1). No significant differences among cultivars were detected at 20, 30, and 40 cm depths, whereas a significant difference was observed only at 10 cm depth. These results indicate that overall soil moisture conditions at harvest were largely comparable among cultivars.

### 2.2. Shoot Characteristics Data

#### 2.2.1. Plant Height, Biomass, and Leaf Area

From the Analysis of variance (ANOVA) of shoot traits (Table 1), plant height, shoot dry weight, and leaf area showed significant differences among cultivars under WW conditions. Under drought conditions, however, significant differences were observed only for plant height and leaf area. In contrast, the root-to-shoot dry weight ratio did not differ significantly among cultivars under either WW or drought conditions.

**Figure 2:**
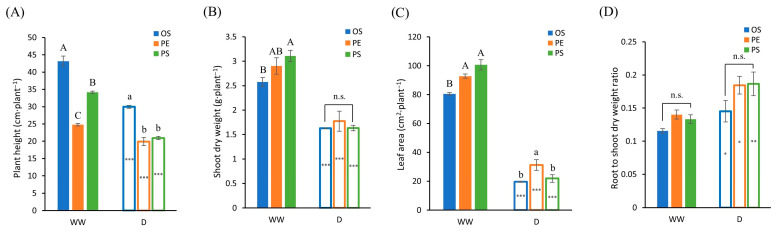
Soybean shoot traits under well-watered (WW) and drought (D) conditions. (**A**) Plant height, (**B**) shoot dry weight, (**C**) leaf area, and (**D**) root to shoot ratio. Different uppercase letters indicate significant differences among cultivars within the WW condition, and different lowercase letters indicate significant differences among cultivars within the D condition. In the figure, n.s. denotes non-significant differences. Asterisks indicate significant differences between WW and D conditions within the same cultivar (*: *p* < 0.05; **: *p* < 0.01; ***: *p* < 0.001). The data were collected in three replicates and were presented as the average ± standard error.

**Figure 3:**
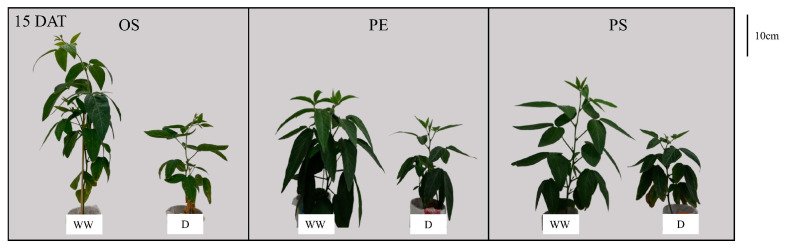
Representative shoot images of soybean cultivars (OS, PE and PS) under well-watered (WW) and drought (D) conditions at 15 days after treatment (15 DAT).

Plant height was reduced in all cultivars under drought compared with WW conditions. Under WW conditions, Osoy (OS) exhibited the greatest plant height, followed by Pungsannamul (PS) and PE529 (PE). Under drought conditions, OS consistently maintained the greatest height, whereas no significant differences were observed between PS and PE. When comparing water treatments within each cultivar, plant height decreased by 31% in OS, 20% in PE and 39% in PS, indicating that PS exhibited the greatest reduction under drought, whereas PE showed the smallest reduction (Figure 2A and Figure 3).

Shoot dry weight differed significantly among cultivars under WW conditions. PS exhibited a significantly higher shoot dry weight than OS, whereas PE did not differ significantly from either PS or OS. In contrast, no significant cultivar differences were detected under drought (Table 1). When comparing WW and drought treatments within each cultivar, shoot dry weight decreased by 37% in OS, 39% in PE, and 47% in PS. As the magnitude of reduction followed the same cultivar ranking observed for shoot dry weight under WW conditions (PS > PE > OS), the cultivar differences in shoot dry weight were diminished under drought conditions (Figure 2B).

Leaf area also responded differently to drought among the cultivars. Under WW conditions, PE and PS did not differ significantly, whereas OS exhibited a significantly smaller leaf area than the other two cultivars. Under drought conditions, PE maintained a relatively larger leaf area. The reduction in leaf area from WW to drought was 75% in OS, 66% in PE, and 78% in PS, indicating that PE experienced the smallest reduction (Figure 2C).

The root-to-shoot dry weight ratio did not differ significantly among cultivars within each water treatment (Table 1) but increased significantly under drought compared with WW conditions across all cultivars (Figure 2D). This indicates that root biomass increased proportionally relative to shoot biomass, reflecting enhanced carbon allocation to belowground biomass in response to drought stress.

#### 2.2.2. Photosynthetic Parameters

Under WW conditions, neither net carbon assimilation rate (*A_net_*) nor stomatal conductance to water vapor (*gsw*) showed a consistent cultivar ranking over time, with values fluctuating without a stable cultivar-specific pattern. Under drought conditions, differences among cultivars were observed over time, although these differences were not consistently significant at all measurement points.

**Figure 4:**
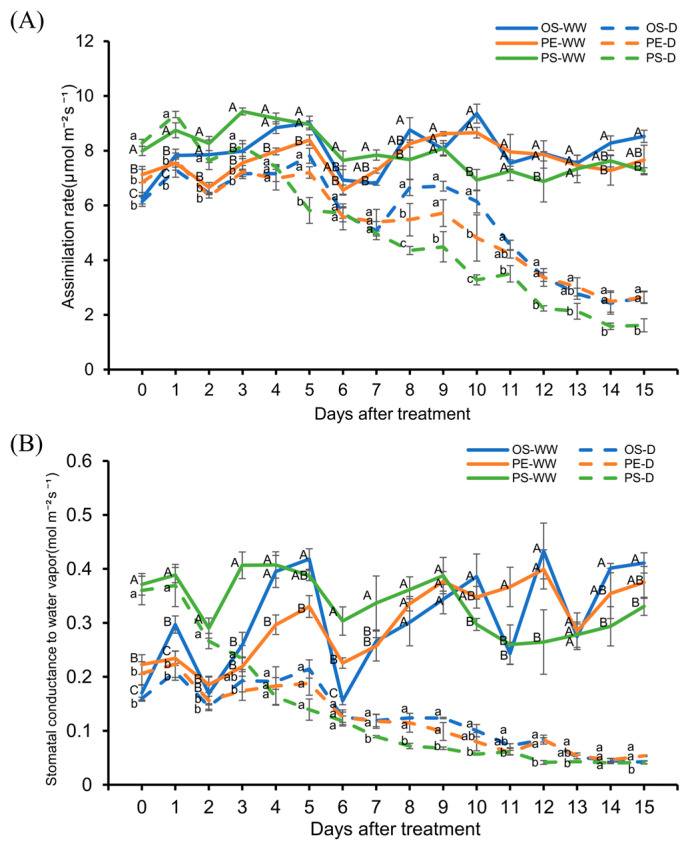
Leaf gas exchange parameters under well-watered (WW) and drought (D) conditions. (**A**) Effect on assimilation rate (*A_net_*), and (**B**) effect on stomatal conductance to water vapor (*gsw*). In each graph, different uppercase letters indicate significant differences among cultivars under WW conditions, whereas different lowercase letters indicate significant differences among cultivars under D conditions.

For *A_net_*, PS exhibited higher values than OS and PE during the early phase of drought treatment (up to 3 DAT), after which no significant differences were detected among cultivars for several days. From 8 DAT onward, however, PS tended to exhibit lower *A_net_* values compared with the other cultivars, and significant differences were observed at multiple later time points (Figure 4A).

A similar pattern was observed for *gsw*. PS showed higher *gsw* values than the other cultivars during the early drought phase (up to 3 DAT), followed by a period with no significant cultivar differences. From 7 DAT onward, PS generally exhibited the lowest *gsw* values among the cultivars, with significant differences detected at several time points (Figure 4B).

### 2.3. Root Characteristics Data

#### 2.3.1. Root Morphological Traits

**Figure 5:**
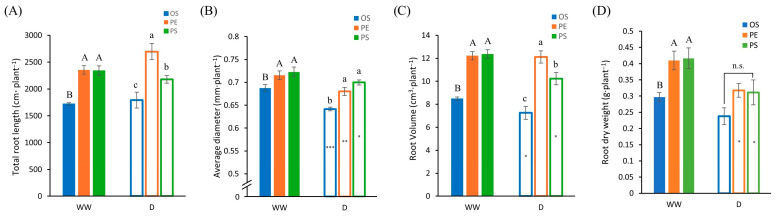
Soybean root traits measured under well-watered (WW) and drought (D) conditions. (**A**) Total root length, (**B**) average diameter, (**C**) root volume and (**D**) root dry weight. Uppercase letters indicate significant differences among cultivars under WW conditions, and lowercase letters indicate significant differences among cultivars under D conditions. In the figure, n.s. indicates non-significant differences. Asterisks denote significant differences between WW and D conditions within the same cultivar (*: *p* < 0.05; **: *p* < 0.01; ***: *p* < 0.001). The data were collected in three replicates and were presented as the average ± standard error.

From the ANOVA results (Table 2), all root traits under WW conditions differed significantly among soybean cultivars, with PE and PS showing significantly higher values than OS across all traits (Figure 5). Under drought conditions, significant cultivar differences were observed for total root length (TRL), average diameter (AD), and root volume (RV), whereas root dry weight did not differ significantly among cultivars (Table 2). Unlike the WW conditions, the ranking pattern among cultivars changed under drought stress, with PE exhibiting the highest values followed by PS and OS for both TRL and RV (Figure 5A,C). In contrast, AD maintained the same cultivar pattern as observed under WW conditions (Figure 5B).

For TRL, no significant differences were detected between WW and drought treatments within each cultivar, indicating that drought did not reduce overall root elongation (Figure 5A). In contrast, AD decreased significantly under drought compared with WW conditions in all cultivars, with reductions of 7%, 5% and 3% in OS, PE and PS, respectively (Figure 5B). RV responses differed among cultivars. RV decreased significantly under drought by 15% and 17% in OS and PS, respectively, whereas no significant change was observed in PE (Figure 5C). As a result, the cultivar ranking pattern shifted under drought, reflecting a relative advantage of PE in maintaining RV, while reductions in OS and PS contributed to the altered pattern. Root dry weight differed significantly among cultivars under WW conditions but showed no significant cultivar differences under drought (Table 2). Comparison between water treatments showed that root dry weight decreased significantly by 22% and 25% in PE and PS, respectively, whereas no significant change was observed in OS, leading to the disappearance of cultivar differences under drought conditions (Figure 5D).

#### 2.3.2. Relative Root Length Distribution per Soil Depth

Relative root length distribution was calculated as the proportion of root length at each soil depth relative to TRL across all soil depths, to assess vertical root distribution within the soil profile. Under WW conditions, no significant differences were detected at the 10 cm and 30 cm depths. At 20 cm depth, PS exhibited a lower relative root length compared with the other cultivars, whereas at 40 cm depth, PS showed the highest relative distribution, indicating a more evenly distributed root system along the soil profile. In comparison, OS and PE showed a greater proportion of roots concentrated in upper soil layers, reflecting shallower rooting patterns under WW conditions.

**Figure 6:**
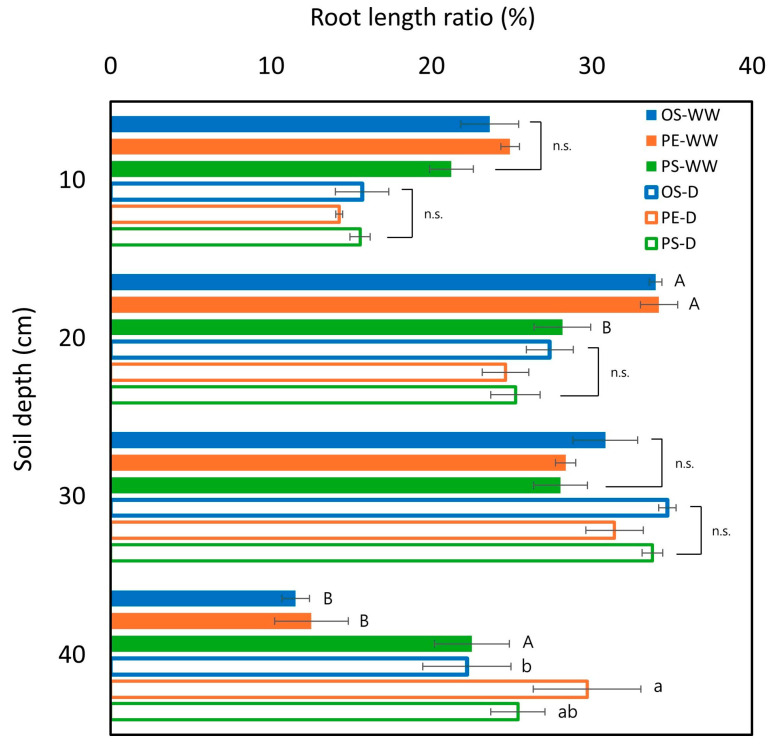
Relative distribution of root length across four soil-depth segments (10, 20, 30, and 40 cm) under well-watered (WW) and drought (D) conditions for the OS, PE, and PS cultivars. Values represent the percentage of total root length contained within each depth segment. Different uppercase letters indicate significant differences among cultivars within the WW condition, whereas different lowercase letters indicate significant differences among cultivars within the D condition. In the figure, n.s. indicates non-significant differences. The data were collected in three replicates and were presented as the average ± standard error.

**Figure 7:**
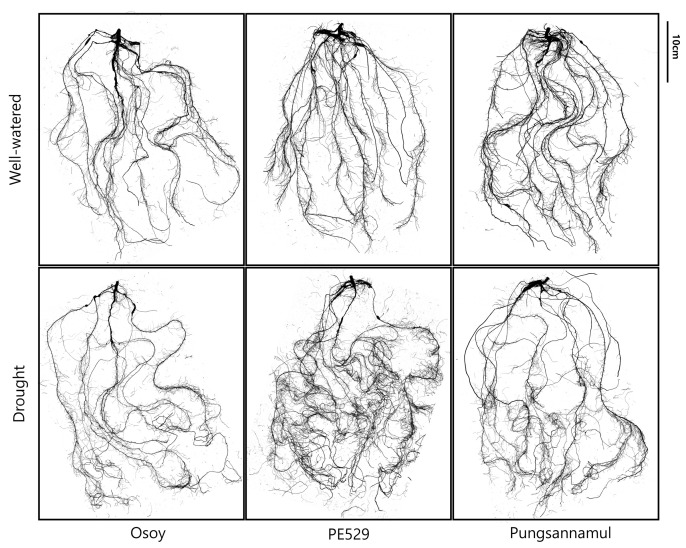
Representative root images obtained from scanned samples of three soybean cultivars grown under well-watered and drought conditions.

Under drought conditions, no significant cultivar differences were observed at depths between 10 and 30 cm. However, at the 40 cm depth, PE exhibited a significantly higher proportion of root length than OS and PS. This observation suggests that PE tended to allocate a greater fraction of its root system to deeper soil layers under drought, which is consistent with its overall favorable root performance under water-limited conditions (Figure 6). A qualitative visualization of the roots of three different soybeans under drought and WW conditions has been illustrated in Figure 7.

### 2.4. Water Use Efficiency

#### 2.4.1. Intrinsic Water Use Efficiency (iWUE)

**Figure 8:**
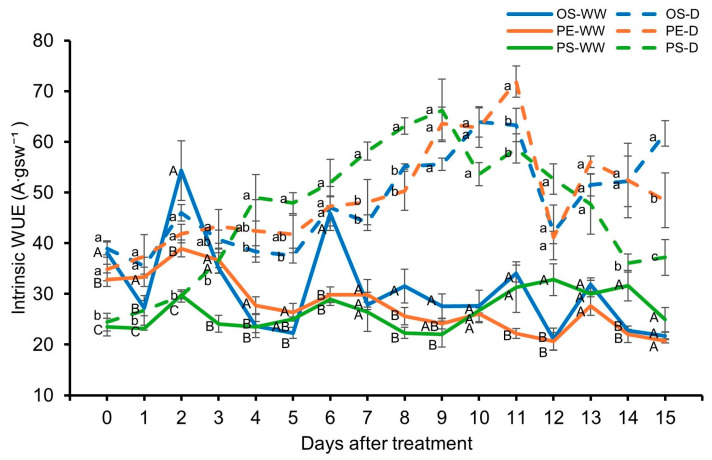
Intrinsic water-use efficiency (iWUE) under well-watered (WW) and drought (D) conditions. Different uppercase letters indicate significant differences among cultivars within the WW conditions, whereas different lowercase letters indicate significant differences among cultivars within the D conditions.

Intrinsic water use efficiency (iWUE) increased under drought conditions compared with WW conditions and generally showed an upward trend as drought progressed. Differences among cultivars were observed over time. PS showed a marked increase in iWUE during the early phase of drought, followed by a decline after 11 DAT, whereas OS and PE tended to maintain relatively more stable iWUE values than PS throughout the drought period (Figure 8).

#### 2.4.2. Whole-Plant Water Use Efficiency (wWUE)

**Figure 9:**
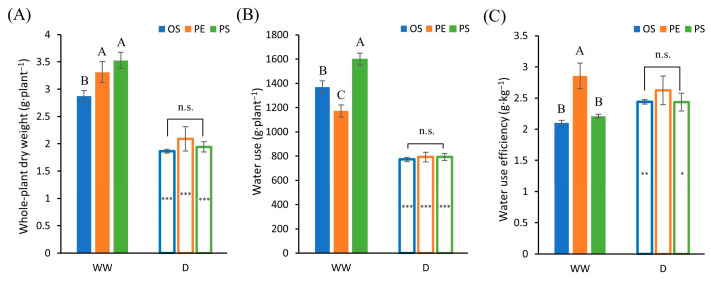
Whole-plant water-use efficiency (wWUE) and its component traits under well-watered (WW) and drought (D) conditions. (**A**) shows whole-plant dry weight, (**B**) shows total water use, and (**C**) shows wWUE derived from these two variables. Different uppercase letters indicate significant differences among cultivars within the WW conditions, whereas different lowercase letters indicate significant differences among cultivars within the D conditions. In the figure, n.s. indicates non-significant differences. Asterisks indicate significant differences between WW and D conditions within the same cultivar (*: *p* < 0.05; **: *p* < 0.01; ***: *p* < 0.001). The data were collected in three replicates and were presented as the average ± standard error.

According to the ANOVA analysis, under WW conditions, whole plant dry weight, water use, and whole-plant water use efficiency (wWUE) were significantly affected among the soybeans. However, all these traits showed non-significant difference under drought conditions (Table 3). Cultivar PS had significantly higher whole plant dry weight and water use compared to cultivar OS under WW condition (Figure 9A,B). As the ANOVA results suggested, no significant differences were observed among cultivars under drought conditions for these three traits. wWUE, calculated as whole-plant dry weight divided by total water use, differed significantly among cultivars under WW conditions, with PE showing the highest wWUE. In contrast, under drought conditions, no significant cultivar differences in wWUE were observed (Figure 9C).

**Figure 10:**
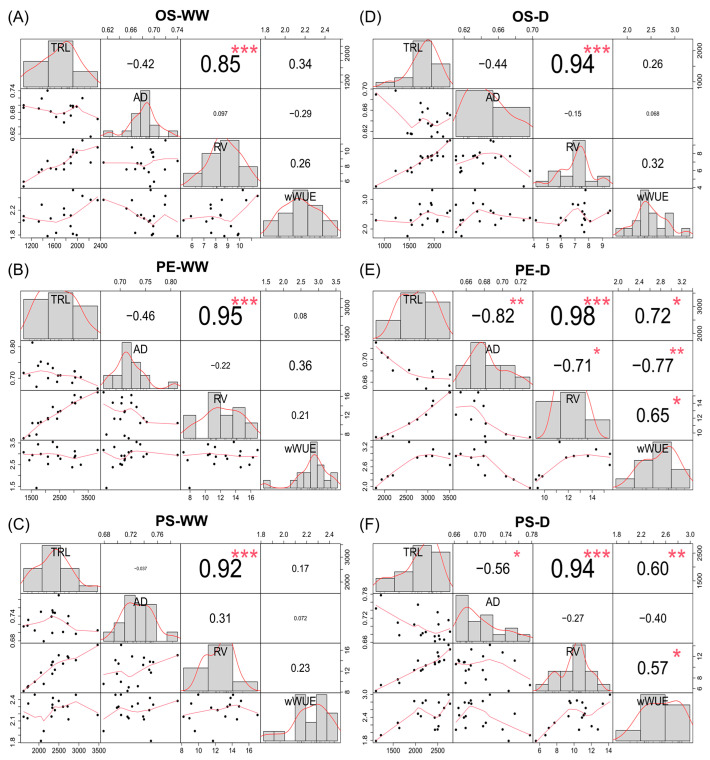
Correlation matrix showing relationships among total root length (TRL), average diameter (AD), root volume (RV), and whole-plant water use efficiency (wWUE) in soybean cultivars under well-watered (WW) and drought (D) conditions. The numbers in each cell represent correlation coefficients, ranging from −1 (strong negative correlation, red) to +1 (strong positive correlation, blue), while values close to 0 indicate little or no correlation (grey/white). The font size of the coefficients is proportional to the absolute value of Pearson’s r, with larger font sizes indicating stronger correlations. (**A**) Osoy under WW, (**B**) PE529 under WW, (**C**) Pungsannamul under WW, (**D**) Osoy under D, (**E**) PE529 under D, and (**F**) Pungsannamul under D. Asterisks indicate the significance levels of the correlation coefficients (*: *p* < 0.05; **: *p* < 0.01; ***: *p* < 0.001).

A correlation analysis was also done to plot the linear relationship between the root traits and the wWUE. Under drought conditions, correlation analyses revealed cultivar-specific patterns (Figure 10). In PE, wWUE was significantly correlated with both TRL (*r* = 0.72), AD (*r* = −0.77) and RV (*r* = 0.65). In PS, a significant correlation was observed between wWUE and TRL (*r* = 0.60) as well as RV (*r* = 0.57), whereas no significant correlations were detected in OS. These results show that the associations between wWUE and root traits under drought differed among cultivars, with PE exhibiting relatively strong and diverse correlations.

## 3. Discussion

Understanding crop performance under soil moisture deficit during the early seedling stage is critical because water stress at this stage can markedly reduce early-season vigor and shoot growth in soybean [26]. In general, plant height and leaf development are considered fundamental processes governing shoot growth and canopy formation, and are known to be highly sensitive to water availability [7,13]. However, the magnitude and pattern of these responses can vary among soybean cultivars, reflecting genotype-specific sensitivity to water deficit [26]. This general pattern was also observed in the present study. Under WW conditions, all cultivars showed normal shoot development; however, under drought conditions, clear cultivar differences appeared in plant height and leaf area. The PE cultivar maintained the largest leaf area and the smallest reduction in plant height under drought, indicating that PE had a superior ability to maintain shoot biomass and photosynthetic potential compared with the other two cultivars under water-limited conditions. A similar pattern was observed in photosynthetic indicators. In the PS cultivar, iWUE decreased sharply as drought continued, whereas OS and PE maintained relatively stable values. In PS, *A_net_* continued to decline even after *gsw* reached a relatively stable level, suggesting a stronger inhibition of photosynthetic metabolism. In contrast, OS and PE exhibited smaller decreases in both *A_net_* and *gsw*, enabling them to maintain relatively stable iWUE throughout the drought period.

Despite these clear cultivar differences in shoot growth and photosynthetic behavior, wWUE did not differ significantly among cultivars under drought, whereas significant differences were observed under WW conditions. This apparent convergence of wWUE under drought likely reflects both physiological and experimental constraints. According to Boyer [27], leaf expansion is among the most sensitive processes to soil water deficit, and once soil water content declines below a critical threshold, leaf expansion rates approach zero, leading to a rapid reduction in growth [28]. Under such conditions, prolonged exposure to water deficit is expected to minimize genotypic differences in biomass accumulation as overall growth becomes uniformly constrained (Figure 9A). In addition, in pot-based systems, roots typically explore most of the available soil [28], as supported by the observation that roots were distributed throughout the soil profile across all depths in this study (Figure 6). Consequently, total water use per plant under drought did not differ among cultivars, with approximately 800 g of plant-available water per pipe (Figure 9B). Nevertheless, only PE maintained significant correlations between wWUE and all root traits under drought, suggesting that PE employed a root-based water-use strategy even under stress conditions.

Roots are the primary plant organs that perceive and respond to changes in soil moisture, and early root traits have therefore been suggested as useful indicators for evaluating cultivar-specific drought responses [6,29]. Analysis of root length distribution by soil depth showed distinct cultivar-dependent patterns under WW conditions (Figure 6). PS exhibited a relatively uniform distribution of root length across soil layers, whereas OS and PE showed a greater proportion of roots concentrated in the upper soil layers. Considering that PS had the highest water use under WW conditions, it is possible that surface soil moisture was depleted more rapidly in this cultivar in the pot environment, which in turn may have induced deeper rooting into the 40 cm depth layer, where more water remained available. This pattern aligns with previous reports showing that roots tend to proliferate in deeper soil layers when surface soil moisture becomes limited, enabling plants to access relatively available water at greater depths [30,31]. In contrast, the relatively lower water use observed in OS and PE under WW conditions may have reduced the necessity for deep rooting.

Under drought conditions, root distribution patterns differed from those observed under WW conditions, with drought treatment associated with a greater relative allocation of roots to deeper soil layers. This shift corresponded with the comparatively higher soil water content observed at the 40 cm depth (Figure 1), suggesting that roots may have proliferated into deeper layers in response to relatively greater water availability in the subsoil. Notably, PE showed a higher proportion of roots at the 40 cm depth compared with OS and PS. This pattern suggests that PE may have maintained a greater capacity for root extension into deeper soil layers under water-limited conditions. Access to subsoil water through deep rooting has been widely reported as an important component of drought adaptation in crops [29,30]. However, deep root systems can also require substantial photosynthate to maintain a large root biomass, which may, in some cases, reduce resources available for shoot growth and yield [31]. Accordingly, a larger or deeper root system is not necessarily advantageous under all drought scenarios, and drought adaptation is more appropriately interpreted through integrated evaluation of multiple root and shoot traits. In addition, the stronger correlation between root traits and wWUE observed in PE suggests that variation in root morphology may have contributed more consistently to individual differences in biomass production under drought in this cultivar (Figure 10). When considered together with shoot growth, photosynthetic traits, wWUE and the root responses observed in PE suggest a more coordinated physiological adjustment to drought relative to the other cultivars.

In the present study, the PE cultivar carries a mutation in the *FAD2-1A* gene that confers elevated oleic acid content; however, *FAD2-1A* is predominantly expressed in developing seeds. Therefore, the fatty acid composition of PE seeds does not necessarily reflect fatty acid composition in vegetative tissues [32]. Furthermore, because PE was developed through ethyl-methanesulfonate (EMS) mutagenesis, the possibility of additional unintended genetic changes beyond the *FAD2-1A* mutation cannot be excluded. EMS mutagenesis is known to induce numerous point mutations across the genome, potentially affecting not only genes related to fatty acid metabolism but also other loci associated with drought responses [33]. Therefore, these considerations suggest that the relatively favorable performance of PE under drought conditions cannot be attributed solely to its elevated oleic acid content, but is more likely the result of complex interactions among multiple physiological and genetic factors.

Meanwhile, the PE cultivar exhibited distinguishing characteristics in water use traits in addition to its oleic acid content. Under WW conditions, PE showed the highest wWUE among the three cultivars (Figure 9). This difference was largely attributed to genotypic variation among cultivars in water use. Indeed, PE exhibited the lowest water use under WW conditions, which resulted in a comparatively higher wWUE. Previous studies have commonly defined drought tolerance as the ability to survive under limited soil water availability [34]. In this context, genotypes with lower total water use are considered to have an advantage, as they deplete soil water more slowly and can therefore better endure periods of drought [35]. Taken together, these findings suggest that the relatively low water use characteristics of PE may have provided a physiological basis that conferred an advantage under drought conditions. Therefore, although functional soybean cultivars differing in fatty acid composition were evaluated under drought stress, it is difficult to attribute the observed drought responses directly to fatty acid composition alone. Rather than aiming to establish direct causal links between specific fatty acid traits and drought tolerance, this study emphasizes a comparative, whole-plant level analysis of agronomic and physiological responses among functional soybean cultivars developed through different breeding pathways under drought stress.

The soybean cultivars used in this study (PS, OS, and PE) were chosen based on their shared genetic background and contrasting fatty acid compositions. These lines represent recently developed breeding materials that have not previously been subjected to detailed drought phenotyping, particularly during early growth stages. Therefore, examining their physiological and root responses under drought conditions provides insights into cultivar-specific adaptation. Despite these considerations, the present study was limited to the vegetative stage, and drought responses were primarily evaluated based on biomass-related traits and water use efficiency. Although shoot and root biomass provide important insights into early-stage drought adaptation, greater biomass does not necessarily translate into higher grain yield under field conditions [31,35]. Early deep rooting and favorable root architectural development may enhance access to subsoil water and influence subsequent root distribution, potentially contributing to plant function during reproductive stages under water-limited environments [36,37,38]. In addition, conservative water-use patterns, higher water-use efficiency, and stable photosynthetic activity during early growth may help conserve soil moisture and mitigate water deficits during flowering and seed-filling stages [39,40]. To more accurately assess drought-adaptive potential in soybean cultivars, future studies should extend evaluations to reproductive stages, incorporate field-based trials, and integrate yield-related traits with physiological and water-use parameters. Moreover, this study focused on phenotypic and physiological responses, and the molecular mechanisms underlying drought adaptation among soybean cultivars with different fatty acid compositions were not investigated. How these factors are integrated in cultivar-specific drought responses during early growth remains to be further clarified. Although fatty acid metabolism is known to play important roles in membrane stability and stress signaling [41], its contribution to cultivar-level differences in drought responses is still not fully understood. Therefore, further studies integrating lipid profiling with physiological and molecular analyses are needed.

## 4. Materials and Methods

### 4.1. Plant Materials and Growth Conditions

Three soybean cultivars with contrasting seed fatty-acid compositions were used in this study. ‘OS’ was developed by crossing ‘Daepung’ with ‘PE2166’, an EMS–induced mutant line of Pungsannamul identified for elevated ω-3 fatty acid (ALA) concentration [42]. ‘PE’ is an EMS–induced mutant line derived from the PS genetic background and carries a mutation in the *FAD2-1A* gene, resulting in elevated OA content compared with the wild type [43,44]. The wild-type cultivar ‘PS’ was used as the control cultivar (Table 4). The soybean cultivars examined in this study were chosen because they share a similar genetic background while differing in their fatty acid composition.

Plants were grown in a plant growth chamber (JSPG-1500C; JS Research Inc., Gongju-si, Republic of Korea) maintained at 28/22 °C (day/night) with an average relative humidity of 65% and illuminated by a mixed halogen and red/blue LED source (R:B = 2:1) delivering 20,000 lux at the canopy with a 14 h light/10 h dark photoperiod. After germination on Petri dishes, seedlings were transplanted into individual rooting pipes and watered daily with 100 mL per pipe. A nutrient solution (High Grade S; Hyponex, Osaka, Japan) diluted 1:1000 (*v*/*v*) was applied in place of water once every three days.

### 4.2. Soil Preparation

#### 4.2.1. Soil Properties and Determining Container Capacity in Rooting Pipes

The soilless medium used for this experiment contained 4% zeolite, 68% cocopeat, 7% perlite, 6% rough stone, 14.73% pittMoss, 0.201% fertilizer, 0.0064% wetting agent, and 0.005% pH modifier (Baroker; Seoul Bio Co., Eumseong, Republic of Korea). Samples of the soilless medium were saturated with water and then allowed to freely drain for 24 h to reach container capacity. The container-capacity mass was recorded, after which the samples were oven-dried at 80 °C until a constant mass was reached (JSOF-150; JSR, Gongju-si, Republic of Korea). Maximum soil water holding capacity (SWHC) was calculated as the difference between the container-capacity mass and the oven-dry mass. The target mass for each pipe was then set to (pipe mass + dry soil mass) + (water mass at maximum SWHC).

#### 4.2.2. Water Retention Curve

**Figure 11:**
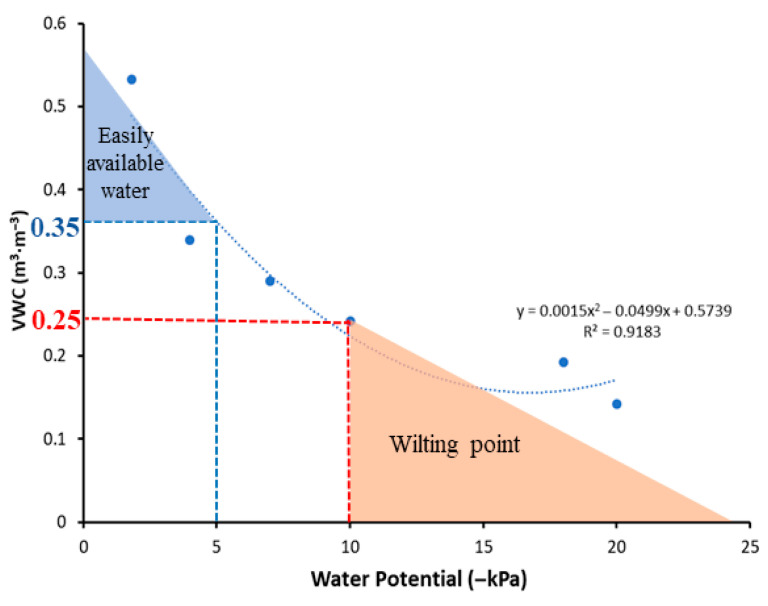
Water retention curve of the soilless medium obtained from tensiometer measurements, showing the relationship between volumetric water content and soil water potential. The six data points represent the measured VWC values at different soil water potentials. The red line indicates the onset of the wilting point, and the blue line represents the upper threshold of easily available water.

Since the physical properties of soilless substrates vary considerably depending on the proportions of peat, coir, perlite, and other components, it is necessary to establish a substrate-specific water retention curve (WRC) to precisely quantify their unique water-air relationships [21]. Although the WRC is commonly derived by fitting a nonlinear van Genuchten model [46], obtaining continuous and high-precision moisture characteristic data is difficult without specialized equipment such as a soil moisture release curve measuring instrument (Hyprop; METER Group, Pullman, WA, USA) system, particularly for peat-based substrates. Thus, in this study, the van Genuchten model could not be applied reliably. Instead, an empirical WRC was constructed by experimentally pairing tensiometer-measured matric potentials with gravimetrically determined water contents across multiple moisture levels (Figure 11).

In this substrate, the measured water contents corresponding to −5 kPa and −10 kPa were VWC of 0.35 m^3^∙m^−3^ and VWC of 0.25 m^3^∙m^−3^, respectively. These matric potentials align with the upper boundary of easily available water and a near-wilting condition, as described in the Introduction. Therefore, the present study defined VWC of 0.35 m^3^∙m^−3^ (−5 kPa) as the WW threshold and VWC of 0.25 m^3^∙m^−3^ (−10 kPa) as the drought threshold.

### 4.3. Data Collection

#### 4.3.1. Soil Data Collection

**Figure 12:**
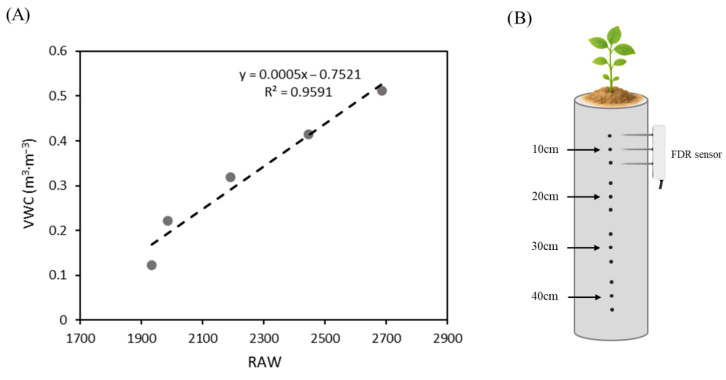
(**A**) Calibration curve of the FDR sensor relating raw sensor values (RAW) to volumetric water content (VWC). Five calibration points were used to derive the linear regression. (**B**) Schematic diagram of the rooting pipe system showing sensor insertion points at 10 cm intervals along the rooting pipe for soil water monitoring.

The rooting pipes were made of polyvinyl chloride with an inner diameter of 8.3 cm and a height of 50 cm. To enable FDR sensor (Teros 12; METER Group, USA) measurements of VWC, side holes (0.6 cm inner diameter) were drilled as follows: at four designated depths (10, 20, 30, and 40 cm), three holes were drilled in a single vertical line, each spaced 10 cm apart, such that the middle hole was positioned at the designated depth (Figure 12B). A plastic liner (50 cm length, 0.05 mm thickness, 8.4 cm inner diameter) was placed inside each pipe to facilitate the extraction of the intact soil column and soybean root system at harvest. To minimize soil evaporation, the upper opening of each pipe was covered with plastic film and the side holes were sealed with label tape, except during FDR measurements. The weight of each pipe (including the plastic liner and label tape) was recorded daily following the measurement of VWC using FDR sensors. After VWC measurements, each pipe was weighed to quantify daily water use and subsequently watered back to 100% SWHC under WW conditions. This procedure continued until plants reached the second trifoliate (V2) stage, at which point drought treatment was imposed.

During the drought period, control pipes were maintained at 100% SWHC each day through gravimetric watering, whereas drought-treated pipes received no irrigation. Harvesting was initiated when any one of the three cultivars reached a soil water content below 0.25 m^3^∙m^−3^ across all monitored depths, a threshold commonly used to indicate drought conditions in soilless substrates (Figure 11). To confirm whether the VWC for all three cultivars was similar among all three depths of measurement, the VWC graphs on the last day of the experiment, i.e., at harvest, were quantitatively illustrated in Supplementary Figure S1. Figure S1 showed that the VWC of all three cultivars was non-significant among each other at all three depths of measurement, except for the 10 cm depth, where OS had significantly higher VWC than the PE and PS. At this point, all pipes were harvested simultaneously to ensure comparable drought exposure among cultivars. Whole-plant water use from the first trifoliate (V1) stage to harvest was calculated following a previously described approach [47].

Water use (g·plant^−1^) = [total water added to each pipe from V1 stage to harvest] + (starting pipe mass − harvest pipe mass) + (whole-plant fresh biomass at harvest) (1)

FDR readings were recorded twice daily at 6 h and 24 h after watering. The 6 h measurement represented short-term changes following irrigation, and the 24 h measurement corresponded to the time immediately before the next irrigation. Measurements were taken throughout the study, beginning at the V1 stage. Prior to data collection, the sensors were calibrated for the substrate, and a substrate-specific calibration curve was applied to convert the raw sensor output (RAW) into VWC, following the manufacturer’s guidelines (Figure 12A).

VWC (m^3^∙m^−3^) = 0.0005 × RAW − 0.7521 (2)

#### 4.3.2. Shoot Data Collection

Leaf gas exchange was measured daily starting at the V2 stage. Measurements were initially taken on the middle leaflet of the trifoliate of the V2 stage, and, at 4-day intervals, the measurement was shifted upward to the middle leaflet of the next newly expanded trifoliate leaf. The *A_net_* and *gsw* were measured simultaneously using a portable photosynthesis system (LI-6800; LI-COR, Lincoln, NE, USA) under controlled conditions, and iWUE was calculated from these two traits (Table 5):

(3) $$Intrinsic\;water\;use\;efficiency=\frac{Assimilation\;rate}{Stomatal\;Conductance\;to\;water\;vapor}.$$

The leaf chamber area was set to 3 cm^2^, with a light intensity of 300 µmol m^−2^ s^−1^, and other chamber parameters were adjusted to match ambient growth chamber conditions. To compare leaf expansion rates among cultivars over the same developmental period, measurements were conducted on a single newly emerging trifoliate leaf. The uppermost trifoliate leaf that appeared 5 days before harvest was identified and tracked, and its leaf area was recorded on the harvest day using an RGB digital camera. Leaf area was then calculated using a leaf image analysis system (WinDIAS; Delta-T Devices, Cambridge, UK). Plant height was recorded immediately after cutting the shoot at the soil surface at harvest. Shoots were then oven-dried at 60 °C until constant mass to obtain shoot dry weight.

#### 4.3.3. Root Data Collection

At the end of the experiment (15 DAT), all plants were cut at the soil surface, and plant height was recorded immediately after harvest. Root samples were carefully separated from the soil, thoroughly washed under tap water, and divided into four equal sections corresponding to soil depths of 10, 20, 30, and 40 cm. Each section was individually stored in plastic bags containing a small amount of water to prevent root drying. Subsequently, a scanner (Expression 12,000XL; Epson, Nagano, Japan) was used to acquire 2D root images. For scanning, a transparent plastic tray (40 cm long × 30 cm wide) was placed on the scanner, and clean tap water was added to the tray. The washed root samples were carefully arranged in the tray, and images were captured when the entire root sample floated on the water surface. Root traits were analyzed using root analysis program (WinRHIZO Pro; Regent Instruments, Inc., Québec City, QC, Canada) (Table 6). Root samples were pat dried and then weighed to determine fresh biomass, then oven-dried at 60 °C until a constant weight was achieved. Final shoot and root dry biomass were recorded for each sample. The root-to-shoot dry biomass ratio was calculated as the ratio of root dry biomass to shoot dry biomass. wWUE was calculated:

(4) $$Whole\text{-}plant\;water\;use\;efficiency=\frac{Whole\text{-}plant\;dry\;weight}{Water\;use}.$$

### 4.4. Experimental Design and Statistical Analysis

The experiment was conducted using a factorial design with two irrigation treatments (daily watering to 100% of maximum SWHC and no irrigation) and three soybean cultivars (OS, PE, PS), arranged with three replicates and six plants per replicate, for a total of 108 pipes. The pipes were placed on a custom-designed stand and were randomized daily after weighing and measuring VWC.

All statistical analyses were conducted using R software (version 4.5.1; R Core Team, Vienna, Austria). ANOVA was performed separately for each irrigation treatment to evaluate differences among cultivars. Following ANOVA, Duncan’s multiple range test was applied to compare cultivars among the treatments. Comparisons between irrigation treatments were conducted within each cultivar using Student’s *t*-test. Correlation analyses between wWUE and root traits were conducted using Pearson’s correlation coefficients. Statistical significance was determined at *p* < 0.05.

## 5. Conclusions

The three soybean cultivars examined in this study exhibited distinct patterns of physiological and morphological responses to drought stress during early growth. PE showed a tendency to maintain deeper root distribution, greater root presence at lower soil depths, and relatively stable photosynthetic performance under water-limited conditions, suggesting a more coordinated adjustment of shoot and root functions. In contrast, OS and PS showed weaker deep root development and reduced physiological stability under drought, despite their vigorous growth under WW conditions. Although the cultivars differ in fatty acid composition, the observed drought responses cannot be attributed solely to variation in fatty acid profiles and are more likely associated with broader physiological characteristics and breeding-related genetic backgrounds. From an agronomic perspective, these results indicate that PE may possess traits favorable for maintaining whole-plant function under limited water availability, whereas OS and PS may require more careful irrigation management during early growth stages. However, this study was conducted under controlled conditions during the vegetative growth stage, and physiological responses at this stage do not necessarily translate directly into yield performance under field conditions. Therefore, the responses identified in this study, such as the deeper root development, smaller reduction in plant height, leaf area and root volume, and more stable photosynthesis observed in cultivar PE, may serve as meaningful physiological indicators for selecting functional soybean cultivars and for designing future studies targeting drought environments in field trials at later growth stages.

## Figures and Tables

**Table 1:** Analysis of variance (ANOVA) for shoot traits of soybean cultivars under well-watered and drought conditions.

Traits	Well-Watered			Drought		
	*df*	*F* Test	*p* Value	*df*	*F* Test	*p* Value
Plant height	2	194.40	<0.001 ***	2	91.62	<0.001 ***
Shoot dry weight	2	4.60	0.0147 *	2	0.83	0.444
Leaf area	2	8.48	<0.001 ***	2	3.54	0.038 *
Root to shoot dry weight ratio	2	3.13	0.0527	2	3.12	0.054

Asterisks indicate levels of statistical significance (*: *p* < 0.05; ***: *p* < 0.001).

**Table 2:** Analysis of variance (ANOVA) for root traits of soybean cultivars under well-watered and drought conditions.

Traits	Well-Watered			Drought		
	*df*	*F* Test	*p* Value	*df*	*F* Test	*p* Value
Total root length	2	7.23	0.00184 **	2	11.04	<0.001 ***
Average diameter	2	7.14	0.00196 **	2	17.97	<0.001 ***
Root volume	2	13.49	<0.001 ***	2	20.57	<0.001 ***
Root dry weight	2	6.37	0.00346 **	2	3.079	0.0561

Asterisks indicate levels of statistical significance (**: *p* < 0.01; ***: *p* < 0.001).

**Table 3:** Analysis of variance (ANOVA) for whole-plant dry weight, water use, and water use efficiency in soybean cultivars under well-watered and drought conditions.

Traits	Well-Watered			Drought		
	*df*	*F* Test	*p* Value	*df*	*F* Test	*p* Value
Whole-plant dry weight	2	5.28	0.00837 **	2	1.16	0.322
Water use	2	15.05	<0.001 ***	2	0.26	0.771
Whole-plant Water use efficiency	2	29.45	<0.001 ***	2	0.96	0.391

Asterisks indicate levels of statistical significance (**: *p* < 0.01; ***: *p* < 0.001).

**Table 4:** Fatty acid composition (%) of the three soybean cultivars (OS, PE, and PS). Values represent the relative proportion of individual fatty acids measured in seeds of each cultivar.

Cultivar	Crossing Combination	Fatty Acid Concentration (%)					References
		Palmitic Acid	Stearic Acid	Oleic Acid	Linoleic Acid	α-Linolenic Acid	
OS	PE2166 × Daepung	9.0	3.0	12.0	61.0	15.0	[45]
PE	EMS-induced mutant line of PS	8.5	2.7	49.1	30.2	9.4	[43]
PS	Wild-type	10.7	3.0	28.1	50.4	7.7	[43]

**Table 5:** Description of leaf gas exchange traits measured using LI-6800.

Traits	Description
Assimilation rate	Rate of net CO_2_ uptake by the leaf measured under controlled chamber conditions using the LI-6800 gas-exchange system
Stomatal conductance to water vapor	Degree of stomatal opening regulating water vapor diffusion from the leaf surface, derived from gas-exchange measurements
Intrinsic water use efficiency	Index describing carbon gain per unit stomatal conductance, calculated as the ratio of net CO_2_ assimilation rate to stomatal conductance to water vapor

**Table 6:** Description of root traits quantified using WinRHIZO.

Traits	Description
Total root length	Total length of all root segments detected from scanned root images using WinRHIZO, reflecting overall root system size and soil exploration capacity
Root Volume	Root volume calculated from diameter measurements for all pixels along the root, representing three-dimensional root biomass investment
Average diameter	Mean diameter of all detected root segments derived from image-based diameter measurements, indicating root thickness and structural investment

## Data Availability

The data is included in the manuscript. Further inquiries can be directed to the corresponding author.

## Supplementary Materials

The following supporting information can be downloaded at https://www.mdpi.com/article/10.3390/plants15050714/s1, Figure S1: Volumetric water content at harvest at soil depths in OS, PE, and PS under drought (D) conditions.

## Abbreviations

The following abbreviations are used in this manuscript:

AD	Average diameter
ALA	α-Linolenic acid
*A_net_*	Net carbon assimilation rate
ANOVA	Analysis of variance
DAT	Day after treatment
EMS	Ethyl-methanesulfonate
FDR	Frequency-domain reflectometry
*gsw*	Stomatal conductance to water vapor
iWUE	Intrinsic water use efficiency
OA	Oleic acid
OS	Osoy
PE	PE529
RV	Root volume
SWHC	Soil water holding capacity
TAG	Triacylglycerols
TRL	Total root length
VWC	Volumetric water content
WRC	Water retention curve
wWUE	Whole-plant water use efficiency
WW	Well-watered
