# Supplementary material for: Investigation of Physiological Responses of Different Soybean Cultivars Under Drought Stress
Source: Plants (Basel). 2026 Feb 27;15(5):714. doi: 10.3390/plants15050714 (PMC12987021; doi:10.3390/plants15050714)
Supplement: Supplementary file 1 [file plants-15-00714-s001.zip › plants-4152611-supplementary.pdf]

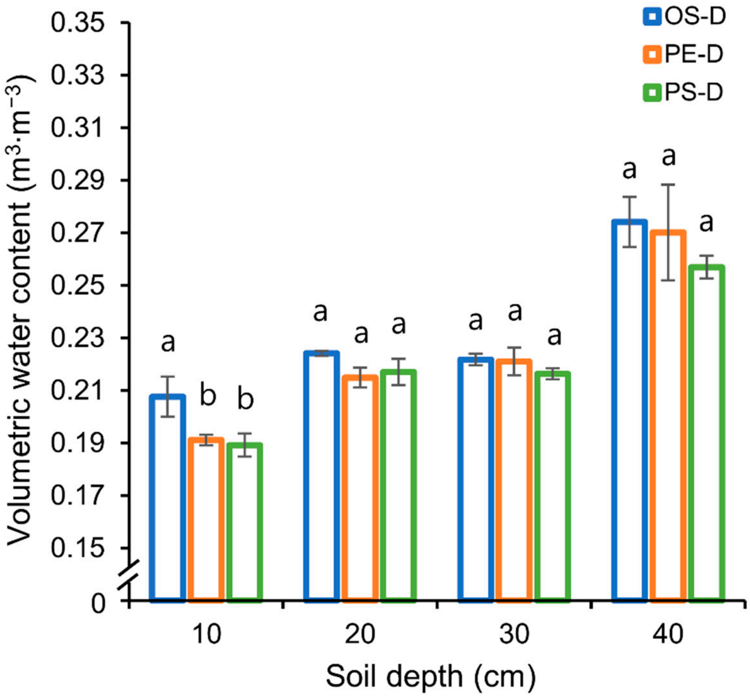

Figure S1. Volumetric water content at harvest at soil depths in OS, PE, and PS under drought (D) conditions. Different lowercase letters indicate significant differences among cultivars at each soil depth. The data were collected in three replicates and were presented as the average ± standard error.
